# Developing an eMental health monitoring module for older mourners using fuzzy cognitive maps

**DOI:** 10.1177/20552076231183549

**Published:** 2023-06-19

**Authors:** Lena Brandl, Lex van Velsen, Jeannette Brodbeck, Sofia Jacinto, Dennis Hofs, Dirk Heylen

**Affiliations:** 1 Roessingh Research and Development, eHealth group, Enschede, The Netherlands; 2 Department of Human Media Interaction, 3230 University of Twente, Enschede, The Netherlands; 3 FHNW School of Social Work, Institute for Consulting, Coaching and Social Management, Olten, Switzerland; 4 Institute for Psychology 27210 , University of Bern, Bern, Switzerland

**Keywords:** eHealth, general: health informatics, general: elderly, medicine: mental health, fuzzy cognitive map, detecting risk situations in eMental health, eMental health

## Abstract

**Objective:**

Effective internet interventions often combine online self-help with regular professional guidance. In the absence of regularly scheduled contact with a professional, the internet intervention should refer users to professional human care if their condition deteriorates. The current article presents a monitoring module to recommend proactively seeking offline support in an eMental health service to aid older mourners.

**Method:**

The module consists of two components: a user profile that collects relevant information about the user from the application, enabling the second component, a fuzzy cognitive map (FCM) decision-making algorithm that detects risk situations and to recommend the user to seek offline support, whenever advisable. In this article, we show how we configured the FCM with the help of eight clinical psychologists and we investigate the utility of the resulting decision tool using four fictitious scenarios.

**Results:**

The current FCM algorithm succeeds in detecting unambiguous risk situations, as well as unambiguously safe situations, but it has more difficulty classifying borderline cases correctly. Based on recommendations from the participants and an analysis of the algorithm's erroneous classifications, we propose how the current FCM algorithm can be further improved.

**Conclusion:**

The configuration of FCMs does not necessarily demand large amounts of privacy-sensitive data and their decisions are scrutable. Thus, they hold great potential for automatic decision-making algorithms in mental eHealth. Nevertheless, we conclude that there is a need for clear guidelines and best practices for developing FCMs, specifically for eMental health.

## Introduction

The loss of a partner is a common occurrence in later life. While most older mourners cope with the loss of their spouse, some (about 10% according to Lundorff et al.^1^) struggle with bereavement and develop prolonged grief.^2,3^ Severe grief symptoms that persist longer than 6 months after bereavement are characteristic of prolonged grief and can result in other mental and physical problems, including depression and cardiovascular problems, and, in extreme cases, suicidal tendencies.^4^ Internet-based interventions have been shown to be effective for treating (prolonged) grief.^5–7^

Some eMental health services combine a web-based self-help program and minimal, but regular therapist contact.^8^ One form of eMental health that blends self-help with professional contact is support on-demand. In support on-demand, where no regular contact with a healthcare professional is planned, the client initiates contact with a professional. These client-initiated contacts are focused on the client's specific needs at that moment, while otherwise following the eMental health service on their own.^9,10^ In settings where no regular contact with a professional is scheduled, eMental health services have the responsibility to refer users to professional human care if the condition of the user deteriorates.^11^ Based on existing mental health safety protocols,^12^ an analysis of professionals’ core competencies in mental health telephone triage,^13^ and in-depth discussions with experts, Tielman et al.^11^ have developed safety protocols for referring users of eMental health services to human care in case of a risk situation. This referral can take the form of an automatic system action. Alternatively, the system can aim at convincing clients to take the initiative in contacting a professional, promoting a self-referral process. Tielman et al.^11^ distinguish two stages in this (self-)referral process: information gathering and decision-making.

The goal of the *information gathering* stage is to identify whether a risk situation exists. In most current eMental health services,^14,15^ a healthcare professional is involved at this stage. For example, in the *Reframe IT* intervention, to reduce suicide risk in secondary students, human caregivers regularly screened the students’ scores on a distress checkup and immediately alarmed the school staff and other appropriate healthcare authorities if necessary.^14^ However, human assessments, such as 24/7 messaging services and screening of distress, are unfeasible for self-help eMental health services that do not have people at their disposal to make such assessments. Alternatively, monitoring whether the user mentions the specific risk (e.g. monitoring suicide ideation) while interacting with the service, combined with routine screening via rating scales, have been proposed^16^ and have shown good results. In self-help eMental health services, the user data collected to identify whether a risk situation exists, such as the user mentioning the specific risk, can be stored and updated in regular measurement intervals while the user interacts with the service. By doing so, the eMental health service collects a profile of the user's mental health, specific to the risk that is relevant in the eMental health service. By updating the variables stored in this profile regularly, continuous monitoring of risk situations is enabled (e.g. Wolters et al.^17^).

In the second stage, the *decision-making*, it is assessed whether any detected risk situation is severe enough to warrant professional intervention. Most routine mental health safety protocols rely on a human professional that combines client data with protocol guidelines.^11^ When involving a human professional to assess risks is not feasible, an eMental health service needs a decision-making algorithm that combines the gathered user information and arrives at actionable advice for the client. Applying Tielman et al.'s distinction of the information gathering and decision-making stages to eMental health services, a mental health user profile and an algorithm that detects and assesses the severity of risk situations emerge as two prerequisites of a monitoring module that aims to deliver the technical infrastructure for support on-demand.

As such, eMental health monitoring modules have much in common with decision support systems (DSSs). In the past two decades, DSSs have become ubiquitous in medical research and in medical care.^18^ The main role of a DSS is to support practitioners in decision-making. According to Papageorgiou et al.^18^, common inference engines used in medical DSSs are rules, Bayesian theory, Bayesian belief networks, heuristics, semantic networks, neural networks, genetic algorithms and other case-specific algorithms, and combinations of these inference mechanisms*.* The authors identify fuzzy logic to be especially promising for medical diagnosis tasks and reasoning. By allowing differing degrees of truth instead of representing something as either true or false, fuzzy logic is a mathematical representation of vagueness and imprecise, uncertain information.^19^ Fuzzy logic can be implemented in medical decision-making using fuzzy cognitive maps (FCMs), a soft computing tool that synergizes fuzzy logic and neural networks.

This article presents a technical infrastructure for support on-demand in an eMental health service for mourning older adults and the construction process of one of the infrastructure's components. This component constitutes a decision algorithm to detect risk situations using FCMs. Background: overview of the monitoring module gives an overview of a monitoring module developed for the eMental health service, consisting of two components. It describes the first component, a (mental health) user profile that serves the purpose of information gathering. The remainder of the Method section outlines the design and execution of a research study to develop the second component, the risk detection algorithm based on the FCM methodology. The resulting FCM and the corresponding algorithm are presented in the Results section, and implications from initial scenario experiments and further results from the research study are discussed in the Discussion section, alongside recommendations for future work.

## Method

The monitoring module described in this article has been developed in the context of LEAVES, a self-help online intervention designed to soothe the mourning process of older adults who lost their spouse.^20^ The service, based on cognitive behavioral therapy, combines psychoeducation about grief, cognitive behavioral exercises for coping with grief, and creating a new life without the spouse with activity suggestions to foster self-care and promote resilience. The monitoring module is intended to help the user reflect about their mood and recommends proactively seeking either professional offline support or reaching out to their support network in times of need when suffering becomes highly disruptive. At the time of writing this article, the effectiveness and technology acceptance of the LEAVES intervention are investigated in separate research efforts, as described in detail in Brodbeck et al.^21^

### Background: overview of the monitoring module

The construction of the first component of the monitoring module, the monitoring user profile, has two phases: (a) an initial risk assessment (IRA) aimed at identifying whether the service is adequate to meet users’ needs at the moment and establishing a baseline for their emotional state and (b) the continuous assessment of users’ emotional state and behaviors to identify risk situations while using the program. Both phases correspond to a questionnaire that measure relevant user parameters for deciding whether recommending the user to seek offline support is appropriate.

The first questionnaire, the IRA, is completed during the introduction of the service to the user. Based on the users’ responses, the service may display recommendations for using the program that, under certain circumstances, suggest seeking further human care to receive adequate support while using the self-help service. These IRA recommendations were categorized into three urgency levels of human help seeking, which translate into more or less pressingly formulated recommendations. Rules formulated by a team of clinical experts determine the urgency level based on the user's risk assessment responses. For instance, the highest level of urgency is triggered when users’ *Suicidality* responses exceed a specific threshold. This recommendation requests users to get into contact with a regional suicide hotline immediately and strongly advises them to confide in a professional or another person they trust. The medium urgency level is triggered when the loss has occurred in the past 12 months, the loss has been violent (accident, suicide, or homicide), and the user has had an inpatient psychological or psychiatric treatment within the last year. The medium urgency level is also triggered if the loss has occurred longer than 12 months ago, but only if users’ *Suicidality* responses exceed a threshold. This *Suicidality* threshold is lower than for the first urgency level. The lowest level of urgency is triggered when the loss has occurred more than 12 months before starting to use the service and the user has either experienced a violent loss or the user has had an inpatient psychological or psychiatric treatment within the last year, or both. Additionally, if the loss has occurred less than 1 month ago, users are given the advice to wait a bit longer before starting the program as the need to deal with practical aspects of the loss and emotional turmoil is common in this period.^22,23^ Except in this case, regardless of the urgency level for the recommendations, the user can start using the program. If no IRA recommendation is triggered, the user is encouraged to start using the service instead.

**Figure 1.**
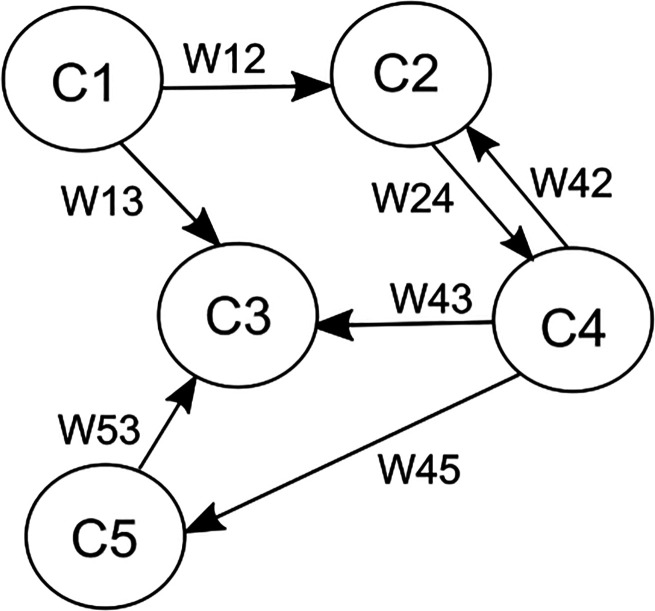
Overview of the monitoring module of an online service for older mourners to recommend seeking offline support whenever advisable.

During the second phase, the continuous risk assessment (CRA) is repeatedly administered and a FCM decision algorithm determines each time whether a *recommendation to seek offline support* is needed. If a risk situation is detected and a recommendation is triggered, the system displays support options, such as a regional telephone hotline and the explicit suggestion to seek human care. Otherwise, the user is encouraged to continue using the service without further recommendations. The CRA is administered for the first time at the end of the user's introduction to the service. Afterwards, it is administered every second week. The first CRA measurement serves as a baseline. Consequently, the FCM decision algorithm is run starting from the second measurement, once the user has used the service for at least 2 weeks. The IRA and CRA represent entries to the user profile for the information-gathering stage, according to Tielman et al.’s model. Figure 1 shows an overview of the different components of the monitoring module. The recommendation to seek offline support is depicted as *Escalate* and the recommendation to keep using the service without taking any further action is depicted as *Do not escalate*.

#### Monitoring user profile

As introduced in the Background: overview of the monitoring module section, the first component of the monitoring module is the user profile, consisting of the initial and the CRA questionnaires. The questionnaires are based on a ranked set of 18 user parameters for monitoring identified by a Delphi study^24^ involving 16 experts in grief and eHealth.

The set included clinical parameters such as *Suicidality* and (*Complicated*) *Grief symptoms*, behavioral and emotional parameters such as *Social isolation* and *Hopelessness*, parameters describing the interaction between the user and the service, such as *Unresponsiveness*, and finally, parameters external to the service, such as the estimation of the user's situation from the perspective of a close one. The 10 highest-ranking parameters from the study were scrutinized for (a) their suitability to detect changes in users’ situation in a biweekly measurement interval and (b) for reliable assessment that uses a minimal number of questions to reduce cognitive demands on the user. A selection of five parameters from the Delphi research was extended with five parameters for the IRA and one parameter for the CRA. Table 1 lists the monitoring parameters assessed in the IRA and in the CRA.

**Table 1.** Summary of the monitoring parameters in the questionnaires for the initial risk assessment (IRA) and the continuous risk assessment (CRA).

**Monitoring Parameter**	**Definition**
Time since loss (IRA)	The number of months since the loss. Measured in four intervals: less than a month, 1–6 months, 7–12 months, and more than 12 months ago.
Violent loss (IRA)	Violent losses considered in the monitoring are accidents, suicide, and homicide.
Recent inpatient treatment (IRA)	Whether or not the user has undergone, in the previous year, a psychiatric inpatient treatment.
Technical skills (IRA)	Lack of digital literacy can impact the users’ motivation to use the service. Basic computer skills are sufficient to use the service.
Crisis detection (IRA and CRA)	The extent to which users have experienced a crisis in the past 2 weeks, meaning that they were impaired in their daily activities due to emotional distress or felt that they cannot cope alone.
Hopelessness (IRA and CRA)	The extent to which users feel negatively about their future and feel that they cannot do anything about it.
Grief symptoms (IRA and CRA)	Intense feelings of grief, such as being stunned or sad due to emotional distress specifically related to the loss, to the extent that users have been impaired in their daily functioning.
Suicidality (IRA and CRA)	The extent to which users have specific plans for a suicide attempt.
Social isolation (IRA and CRA)	The extent to which users feel burdensome towards their social contact and their actual withdrawal behavior.
Therapeutic progress (CRA)	The users’ perception of the extent to which they are making progress in processing their loss.

For each CRA parameter, except for *Suicidality*, two questions were designed. The parameters *Crisis Detection*, *Hopelessness*, *Grief Symptoms*, and *Social Isolation* are completed on a 4-point Likert scale (*Not at all*, *Several days*, *More than half of the days*, and (*Nearly*) *every day*) measuring how frequent they disrupted users’ daily activities in the last 2 weeks. The design of the response options was based on the Patient Health Questionnaire-9 (PHQ-9) that can be used for diagnosing major depression disorder according to the Diagnostic and Statistical Manual for Mental Disorders 5 (DSM-5).^25^*Therapeutic progress* is measured on a 4-point Likert scale ranging from *Strongly disagree* to *Strongly agree*. *Suicidality* follows a two-step approach. The first item, *In the past two weeks, I have considered committing suicide*, serves as a filter. The user can either endorse the statement with *Yes* or respond *No*. Only if the user replied *Yes*, the remainder of the *Suicidality* items is presented to the user. The four conditional Suicidality items were adapted from the Scale for Suicide Ideation (SSI)^26^ and assess the extent to which the user has explicit suicide plans. The complete IRA and CRA questionnaires are included in the Appendix.

### Background: fuzzy cognitive maps

**Figure 2.**
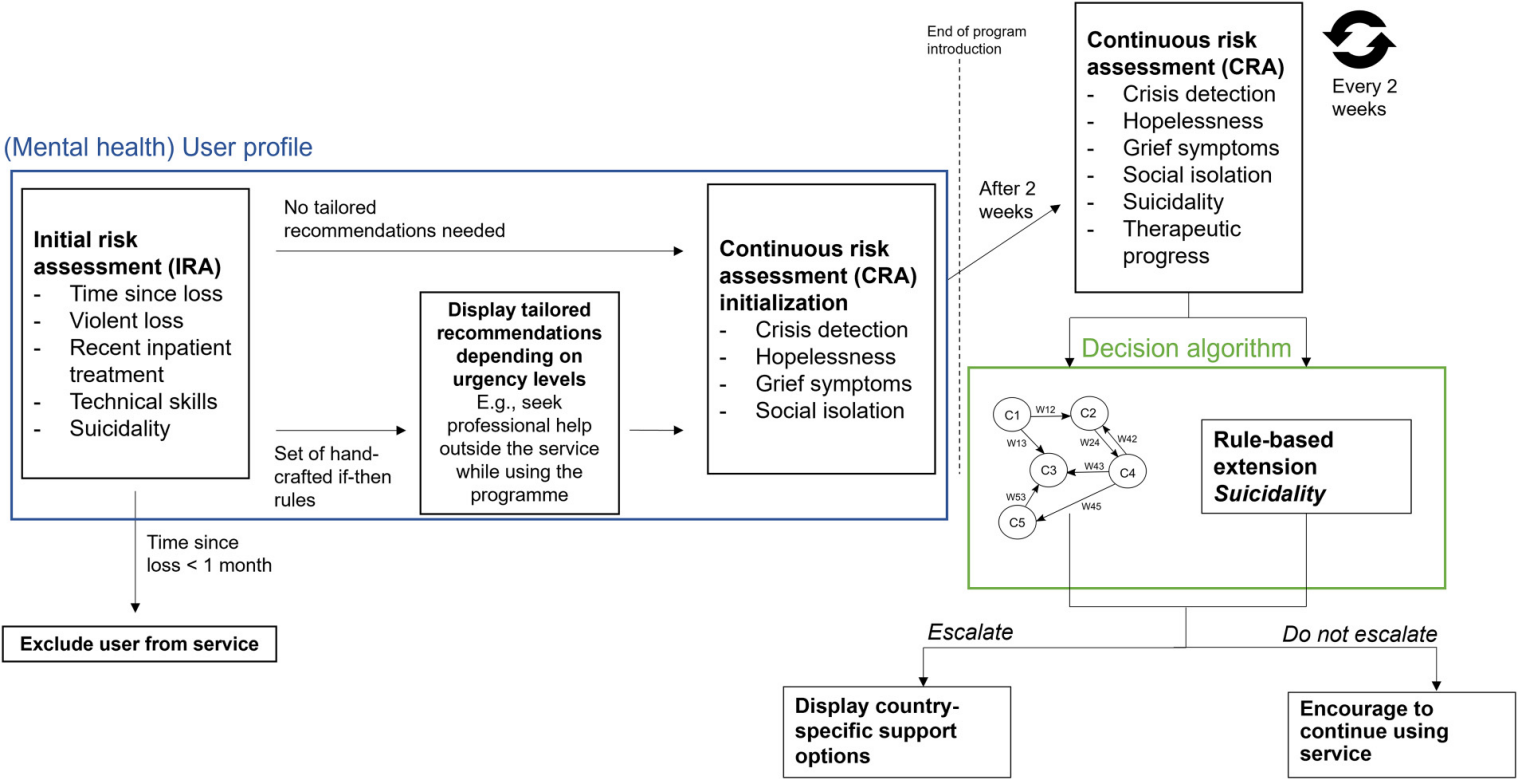
A simple fuzzy cognitive map.

The second component of the LEAVES monitoring module is a risk detection algorithm based on FCMs. FCMs were first proposed by Kosko^27^ as an extension of cognitive maps to model dynamical systems. FCMs are directed graphs that use fuzzy logic to represent causal relations between graph nodes using weighted edges. Figure 2 shows a simple FCM.

In this simple FCM, the causal influence of, for example, concept C1 on concept C2 is indicated with weight W12. There are three types of weights. Weights can either be equal to zero, meaning that that there is no causality between two concepts. Weights larger than zero indicate causal increase (i.e. C2 increases as C1 increases and C2 decreases as C1 decreases if W12 is larger than zero). Weights smaller than zero indicate causal decrease (i.e. C2 increases as C1 decreases and C2 decreases as C1 increases if W12 is smaller than zero). Commonly, the relation between two concepts takes a value in the interval [−1, 1]. −1 corresponds to the strongest causal decrease and 1 to the strongest causal increase. The other values express different levels of influence. The main objective of building a FCM is to predict an outcome state of the FCM concepts by letting them interact with each other according to their weights until their values converge. The concept values at convergence represent the final prediction of the FCM concepts. FCMs have been developed in the context of mental health before. For example, Papageorgiou et al.^18^ developed a FCM-based DSS to diagnose depression in older adults and conclude that one of the strongest points of the FCM-based DSS is that it provides insight into feedback loops between symptoms by making them explicit. For example, one of the symptom concepts identified in the authors’ FCM-based DSS for geriatric depression was *Fatigue*. After constructing the FCM, all connections towards *Fatigue* as well as all ways in which *Fatigue* influences other symptoms in the FCM were known, according to the experts who had helped construct the FCM. That way, the FCM was able to make explicit how an increase in *Fatigue* can bring about an increase or decrease in other symptoms in the model, such as *Depressive mood* or *Indecisiveness*. The configuration of causal relations in FCMs draws on expert knowledge and thereby reflects their reasoning, making them, ultimately, understandable by other human observers, such as healthcare professionals.

### Procedure for developing the FCM decision algorithm

To configure the second component of the monitoring module for the LEAVES intervention, an automatic FCM decision algorithm, interviews with experts in grief were conducted. The interview protocol followed a standard procedure for configuring FCMs with the help of domain experts, described by, for example, Papageorgiou.^28^ The procedure has three main steps. During step 1, key concepts to be included in the FCM are identified. In step 2, the causal relations between the identified concepts are determined. And in step 3, the strength of the relations between concepts is estimated.

Since user parameters for monitoring risk situations in an online grief intervention were available based on existing research,^24^ we used them as the FCM's symptom concepts, corresponding to step 1 in the procedure for constructing FCMs. The monitoring parameters in the IRA and CRA make up the symptom concepts in the current FCM. In addition to the symptom concepts, there are two *outcomes* modeled in the FCM, *Escalate* and *Do not escalate*. They correspond to the two recommendations that the system can give based on the algorithm's calculations. *Escalate* corresponds to the system's *recommendation to seek offline support*, and *Do not escalate* corresponds to *recommendation to keep using the service as is*. This FCM structure is referred to as a competitive FCM (CFCM) because the two outcomes compete with each other, and only one of the two is chosen in the end.^29^

In step 2, experts determine the causal relation between any two concepts in the FCM as either positive, negative, or neutral. The latter case is equivalent to declaring that there is no influence between the two concepts in question. For step 3, commonly, a linguistic variable called *Influence* is declared to represent the strength of relations between concepts (i.e. weights) in fuzzy terms, such as *low*, *medium*, and *high*. The set *T* of qualitative terms that experts in this study used to describe the influence among FCM concepts is: *T*(Influence) = {*Very very low*, *Very low*, *Low*, *Medium*, *High*, *Very high*, *Very very high*}. All qualitative terms occurred either in combination with a positive sign to represent a positive causal relation or in combination with a negative sign to represent a negative causal relation (e.g. −*Very high* or +*Medium*). After these initial three steps to obtain qualitative weights based on expert knowledge, the weights provided by individual experts are aggregated. The process of weight aggregation is described in more detail in the Aggregated FCM weights section.

#### Experts recruitment

For the interviews, eight experts were recruited via the researchers’ professional networks and via snowball sampling. As inclusion criteria, experts were required to have experience with treating or coaching bereaved adults, preferably older adults. Consequently, psychotherapists as well as grief coaches were the primary focus of the recruitment. If a candidate expert expressed interest in the study, the researchers provided them with more detailed information about the research, including the informed consent and a digital copy of the two monitoring questionnaires (IRA and CRA). All participants provided written informed consent. As a token of gratitude, everyone who participated in the study received a gift card amounting to €25.

#### Data collection

The interview protocol was supported by an online, interactive one-on-one session conducted and recorded via Microsoft Teams. Specifically, the interview was designed to ask experts to, based on their professional experience, determine the predictive value of each FCM concept, such as *Social isolation*, in the assessment of the users’ risk situation. In other words, the goal was determining the weights of the FCM concepts in the decision algorithm that identifies risk and triggers recommendation messages to users when further support is needed. Considering the demanding nature of the interview's core task, before the interview, experts were asked to read a brief description of the monitoring module and to read through the monitoring questionnaire items. During the interview, experts were first asked to describe their professional experience with older mourners and to explain to what extent they observe differences between mourners that generally cope well with their loss and those who do not.

**Figure 3.**
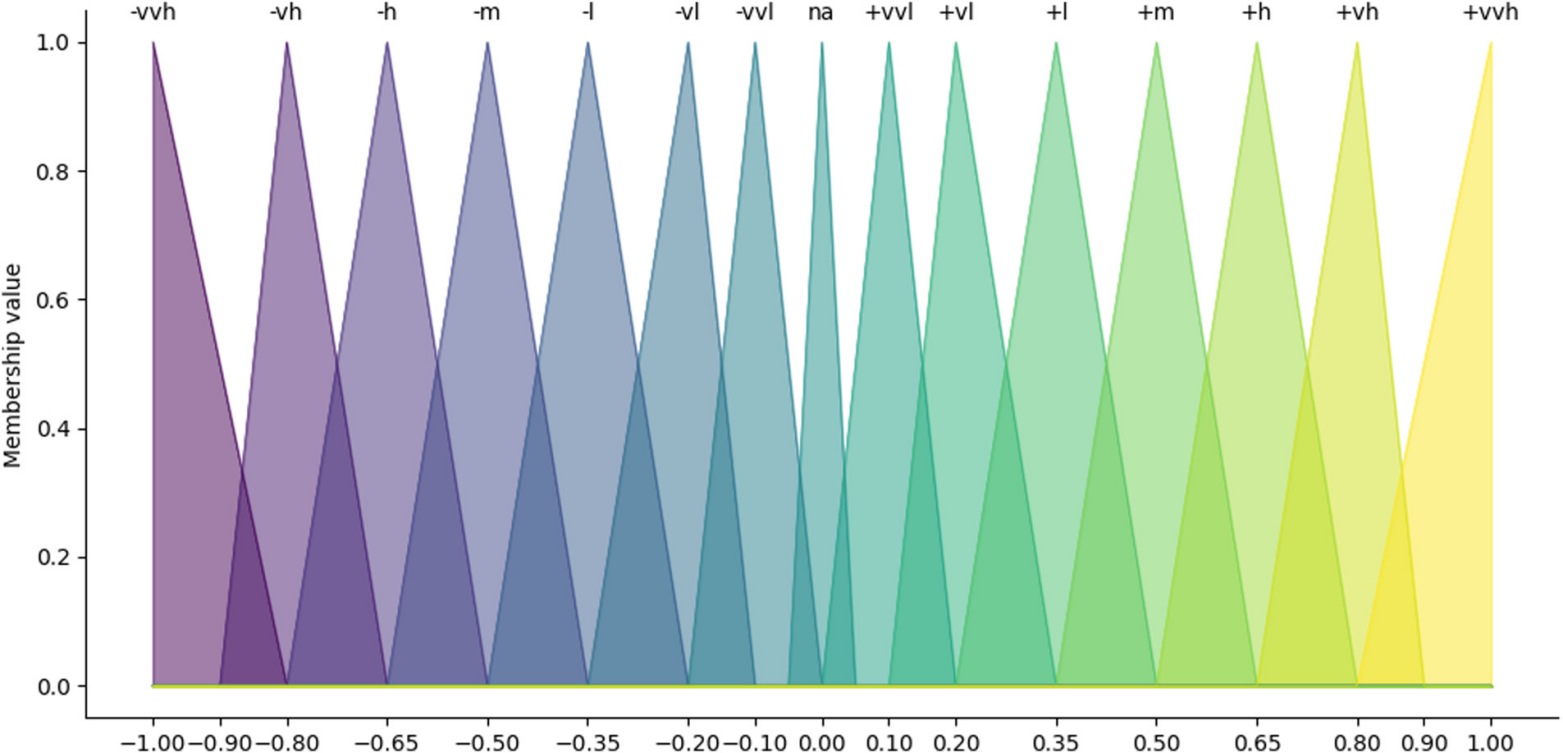
Triangular membership functions of the qualitative terms used by experts to describe the causal relationships in the fuzzy cognitive map between symptoms and outcomes.

**Figure 4.**
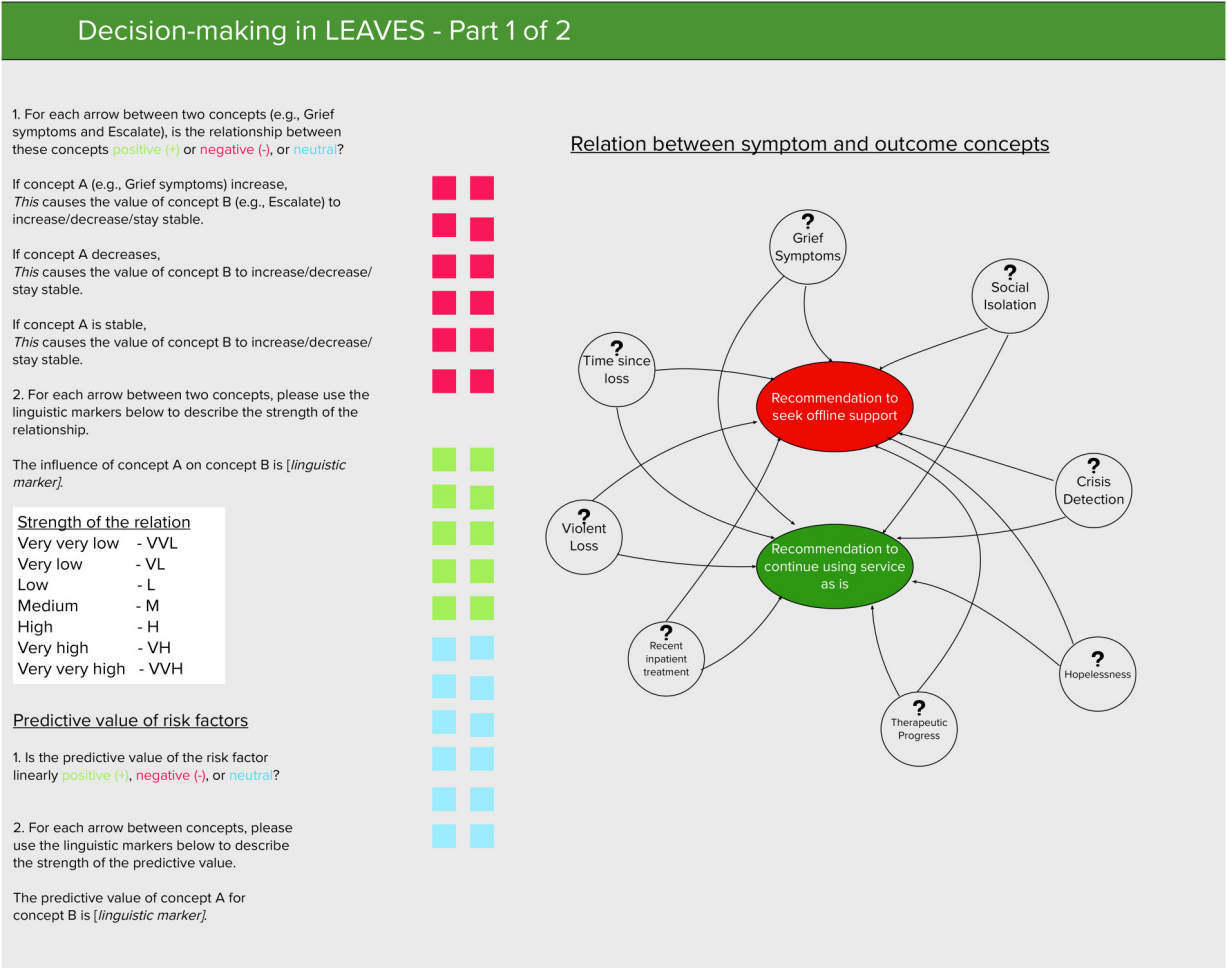
Screenshot of the study materials in Mural.

Then, experts were asked to attribute qualitative values (i.e. weights) from the predetermined *Influence* set *T* ranging from *Very*, *very low* to *Very*, *very high* to each relation between symptom and outcome concepts and among symptom concepts (see Figure 3). Higher weights were indicative of higher risk to develop severe mental health symptoms (i.e. the parameter had a higher predictive value) and lower values were indicative of lower risk (lower predictive value). This part of the one-on-one interviews was conducted with the support of the online collaboration tool Mural.^30^ Mural is comparable to a whiteboard where different content such as text and pictures can be pinned and moved around freely. Via two exercises, experts attributed weights to the relations between the FCM concepts. The first exercise focused on the weights from the eight symptom concepts to the two algorithm outcomes *Escalate* and *Do not escalate*. The second exercise solely focused on relations between symptom concepts. Experts were invited to use colored sticky notes to indicate the relation type between two concepts (e.g. *Hopelessness* and *Social isolation*), which could be positive, negative, or neutral. Our experts attributed weights to all relations between concepts in the first exercise (symptoms to FCM outcomes). Due to time restrictions, in the second exercise, they were asked to assign weights to only the most important relations (symptom to other symptom concepts), according to their professional experience. A snapshot of the Mural board, depicting the visualization of the first exercise, is shown in Figure 4. After experts finished both weighing exercises, the final interview question was to describe how difficult the exercises had been for them. A pilot session was conducted with one psychotherapist. As a result of the pilot, *Suicidality* was excluded from the FCM weighting exercises. The pilot participant explained that considering the serious consequences of active suicidal ideation, *Suicidality* already has relatively clear thresholds for advising a user to seek immediate offline support. Indeed, cutoff scores have readily been used for suicide risk assessment scales to identify people at (immediate) risk for attempting suicide.^31,32^ As a consequence, in the final monitoring module, *Suicidality* is evaluated using decision rules prior to running the FCM. To evaluate *Suicidality* during the CRA, the same escalation threshold is applied as for determining a recommendation of the highest urgency in the IRA.

#### Data analyses

Interviews were transcribed and analyzed via an inductive coding scheme, developed and applied by one researcher, and then verified by a second researcher. Any discrepancies were discussed until agreement was reached. Two examples from the coding scheme are:

- Q2.1CDToOutcome: any explanations and remarks participants make about the relation between the symptom concept *Crisis Detection* and either of the two outcome concepts (*Do not*) *Escalate*.
- FCMModel: any explanations or remarks participants make about using fuzzy cognitive mapping and the proposed FCM in particular, including comments about the choice of (symptom) concepts, model limitations, and suggestions for improving the FCM.

FCMpy,^33^ a library for the Python programming language, was used to combine the qualitative weights (ranging from *Very*, *very low* to *Very*, *very high*) provided by the experts in this study and to transform them into numeric FCM weights. As a preliminary evaluation of the resulting FCM decision algorithm, the obtained weights were tested using four fictitious user scenarios. Scenario testing has a long tradition in FCM research and is regarded as one of the most valuable applications of FCMs.^34^ JFCM,^35^ a library for the Java programming language, was used to simulate the behavior of the FCM based on the four fictitious scenarios. The scenario experiments and their results are described in more detail in the Scenario experiments with aggregated weights section.

## Results

### Expert demographics

In total, eight clinical psychologists participated in the interview study, excluding the pilot session. Three experts were male. Their mean age was 46.5 years, with a standard deviation of 12.76 years. Table 2 summarizes experts’ demographics. All experts had experience as grief coaches and most of them worked with older adults, with 12.5 years of experience on average and a standard deviation of 8.37 years.

**Table 2.** Expert demographics.

	**Age**	**Gender**	**Country**	**Expertise**	**Years of Experience**
Expert 1	29	Female	Switzerland	Psychotherapist (in training) Older adults Grief Depression	4
Expert 2	33	Male	The Netherlands	Clinical psychologist Psychotherapist Researcher Grief Depression	6
Expert 3	41	Female	The Netherlands	Grief coach Lecturer	7
Expert 4	56	Female	The Netherlands	Grief coach Social psychotherapist (in training) Older adults	8
Expert 5	56	Female	The Netherlands	Grief coach Psychotherapist	10
Expert 6	39	Female	Slovenia	Psychotherapist Researcher Suicide	15
Expert 7	53	Male	Switzerland	Clinical psychologist Psychotherapist Lecturer	25
Expert 8	65	Male	The Netherlands	Minister and grief coach	25

### Aggregated FCM weights

Each interview yielded an individual expert's account of the causal relations between symptom (e.g. *Hopelessness*) and outcome concepts (*Escalate* and *Do not escalate*) in the FCM in qualitative terms (e.g. *Low*, *Medium*, and *High*). To develop a FCM, the process of aggregating the input of multiple experts involves four steps: (a) defining membership functions for the qualitative values, (b) applying a fuzzy implication rule, (c) combining the membership functions of individual experts, and (d) defuzzifying the aggregated membership functions to derive a numerical weight for each relation between symptoms and outcomes in the FCM.^33^ The theoretical and mathematical foundation of these steps are explained in more detail elsewhere.^36^

In the first step, triangular membership functions were defined for the qualitative terms used by the clinicians in this study. Triangular membership functions are used in most applications.^28,37^Figure 3 shows the membership functions for the 14 employed qualitative terms in this study: +/− very, very low; +/− very low; +/− low; +/− medium; +/− high; +/− very high; and +/− very, very high. The *na* membership depicts missing values in individual expert's accounts for a specific relationship. Not all experts attributed a qualitative value to every relation because we asked them to only rate those relation between symptom concepts (e.g. *Hopelessness* and Social *isolation*) that they regarded as most important.

Likewise, we chose commonly applied methods for steps two to four; we applied Mamdani's fuzzy implication rule to determine the extent to which each membership function for a specific causal relation was activated (step 2),^38,39^ the algebraic SUM aggregation operation (step 3)^39,40^ for combining the qualitative weights provided by individual experts, and center of gravity (COG) defuzzification to derive a numeric weight for each causal relationship (step 4).^18,41^

**Figure 5.**
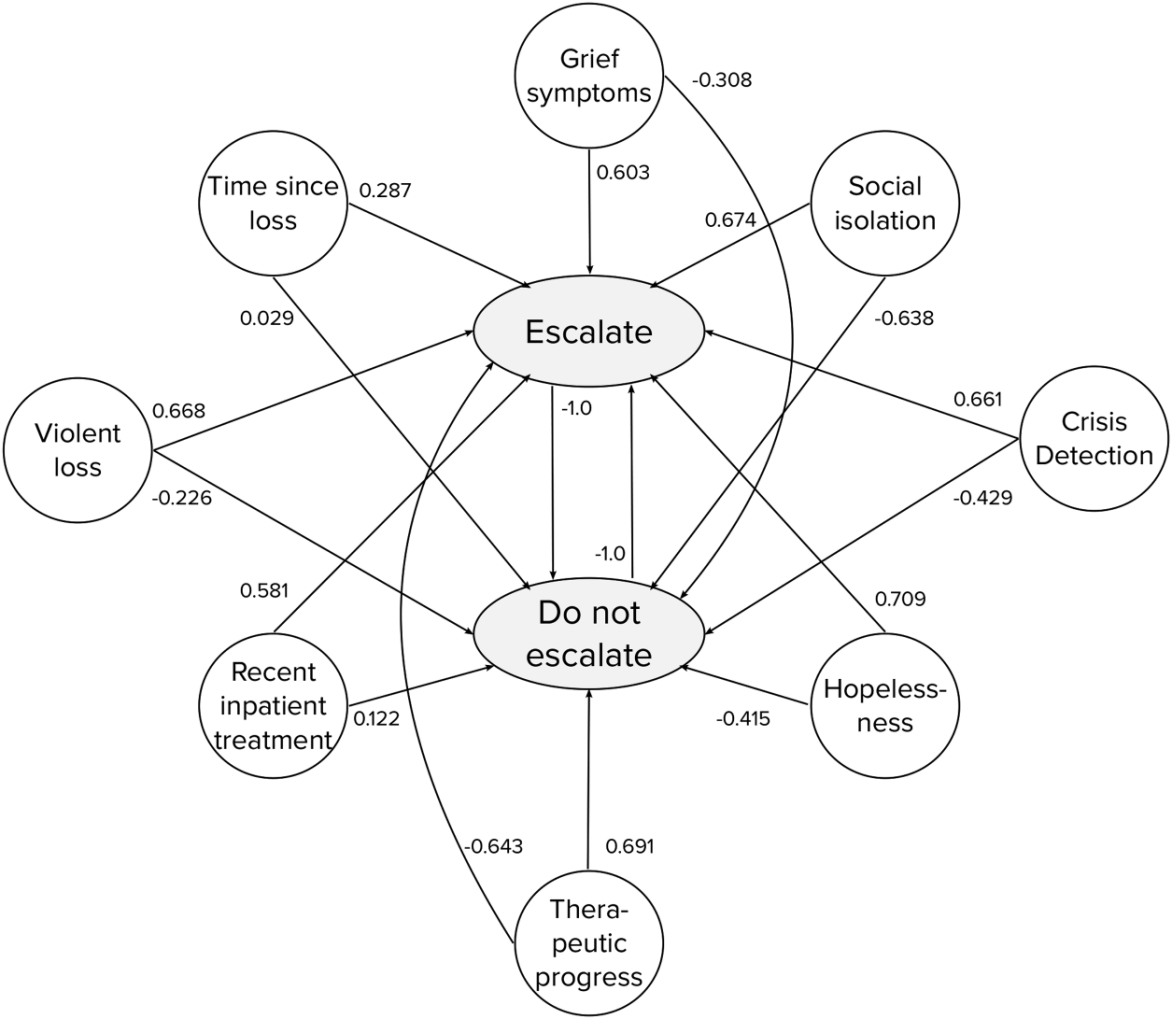
Visualization of the aggregated weights from symptom to outcome concepts.

**Figure 6.**
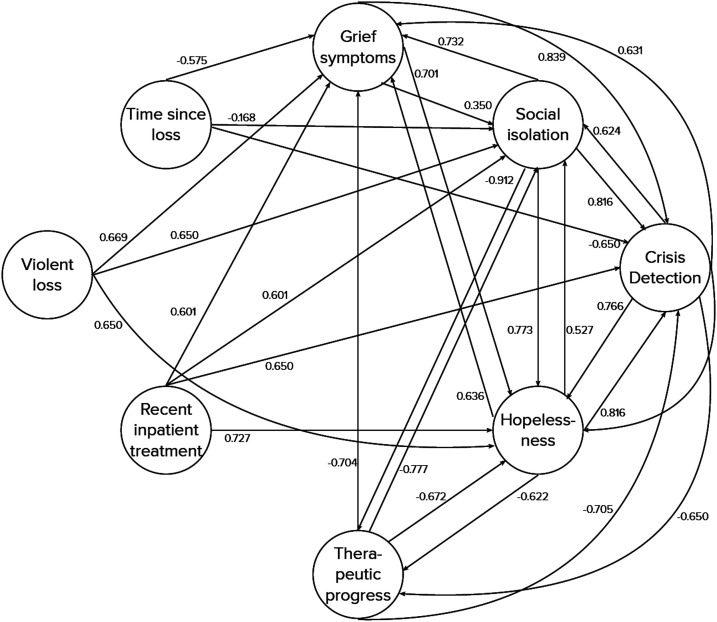
Visualization of the aggregated weights between symptom concepts.

In the resulting weight matrix, due to the competitive nature of this type of FCM which favors one outcome over the other, the relations between the two outcomes *Escalate* and *Do not Escalate* were set to −1. At the same time, the outcomes do not exert any influence on the symptom concepts in the FCM. Hence, all outgoing weights from *Escalate* and *Do not Escalate* were set to 0. Incoming weights from other concepts to *Violent loss*, *Recent inpatient treatment*, and *Time since loss* were also set to 0. There is no logical influence from any of the other symptom concepts on these concepts. The aggregated numeric FCM weights are summarized in Table 3 and displayed in Figures 5 and 6. From the combined weights, a mutually strengthening symptom cluster arises between *Crisis detection*, *Grief symptoms*, *Hopelessness*, and *Social isolation* as indicated by their high and positive relations to each other. They also have strong positive weights towards the outcome *Escalate*. *Therapeutic progress* has a counterbalancing effect on them, indicated by its strong negative weights towards *Crisis detection*, *Grief symptoms*, *Hopelessness*, and *Social isolation*. The three concepts that are included from the IRA questionnaire, *Violent loss*, *Recent inpatient treatment*, and *Time since loss*, all have positive relationships to the *Escalate* outcome. It is notable that experts did not always agree on the type of the relation, i.e. whether the sign of a weight between two symptom concepts or a symptom and an outcome concept was positive or negative. Some experts conceptualized the relation between the two outcome concepts *Escalate* and *Do not escalate* as mutually exclusive, while others did not. While these conceptualization differences did not affect the applied weight aggregation procedures in a mathematical sense (i.e. their principles still applied), this observation shows that FCMs can be used in multiple ways to model the decision-making for detecting risk situations in a grief eMental health service.

**Table 3.** Aggregated fuzzy cognitive map weight matrix.

**To**	**Crisis Detection**	**Hopelessness**	**Grief Symptoms**	**Therapeutic Progress**	**Social Isolation**	**Recent Inpatient Treatment**	**Violent Loss**	**Time Since Loss**	**Escalate**	**Do Not Escalate**
**From**										
Crisis Detection		0.766	0.631	−0.650	0.624				0.661	−0.429
Hopelessness	0.816		0.636	−0.622	0.527				0.709	−0.415
Grief symptoms	0.839	0.701		.0	0.350				0.603	−0.308
Therapeutic progress	−0.705	−0.672	−0.704		−0.777				−0.643	0.691
Social isolation	0.816	0.773	0.732	−0.912					0.674	−0.638
Recent inpatient treatment	0.650	0.727	0.601	.0	0.601				0.581	0.122
Violent loss	0.000	0.650	0.669	.0	0.650				0.668	−0.226
Time since loss	−0.650	.0	−0.575	.0	−0.168				0.287	0.029
Escalate										−1
Do not escalate									−1	

### Scenario experiments with aggregated weights

As a preliminary evaluation of the resulting FCM algorithm, the obtained weights were tested using four fictitious scenarios. As an initial assessment of the utility of the obtained FCM weights, a clinical expert generated four scenarios, each representing the responses of a fictitious user case. Scenario 1 focused on a user that clearly needs support; scenario 2 on a user that can clearly continue using the service; scenario 3 on a user who should seek support, but it does not show obviously from the mourner's questionnaire responses; in scenario 4, the fictitious user should get the advice to continue using the service, but less obviously than in scenario 2.

Before the FCM could be simulated based on the fictitious cases, the monitoring questionnaire responses had to be transformed into numeric values so that they could be used as inputs for the FCM. For each monitoring parameter, a total score was calculated and mapped on the interval [−1, 1]. The CRA questionnaire inquires about the frequency of symptoms in the past 2 weeks. Due to the high prevalence of positive weights towards the outcome *Escalate*, we decided to model the absence of symptoms or very infrequent symptoms as supportive for the outcome *Do not escalate*; hence, we mapped the lowest response options to a negative input value. *Violent loss* and *Recent inpatient treatment* were mapped on either −1 or 1, depending on whether the user endorsed the question. The questionnaire total scores per dimension, corresponding to the four fictitious scenarios, are summarized in Table 4. Since *Suicidality* was removed from the FCM based on the feedback that we received during the pilot, no *Suicidality* scores were included in the scenarios. Instead, the final monitoring algorithm has a rule-based extension that checks users’ suicidality responses for risk situations outside the FCM.

**Table 4.** Summary of fictitious user response scenarios using the aggregated expert-based FCM weights.

**Monitoring Item**	**Scenario #1**	**Scenario #2**	**Scenario #3**	**Scenario #4**
Grief symptoms	5	1	5	4
Social isolation	3	1	2	2
Crisis detection	6	2	5	4
Hopelessness	6	1	2	3
Therapeutic progress	1	5	2	2
Violent loss	0	0	0	1
Recent inpatient treatment	1	0	0	0
Time since loss	1	3	3	1

Scenarios were evaluated using equation (1), Kosko's modified inference equation for FCMs,^28^ which iteratively calculates the values of the concepts in the FCM based on an initial baseline until the model converges. In CFCMs, the outcome concept with the highest value is regarded as the model's outcome. The outcome concepts in this case were *Escalate* and *Do not Escalate*.

Equation (1) shows Kosko's modified scenario evaluation formula:

(1) $$A_{i}^{\left(k+1\right)}=f\left(A_{i}^{\left(k\right)}+\overset{N}{\underset{\;j\neq i;j=1}{\sum }}\;A_{j}^{\left(k\right)}w_{\;ji}\right).$$

A represents the initial state vector of the FCM. *A* is a Nx1 matrix where each of the *N* rows contains the initial value of a concept *C* in the FCM. In this study, the initial concept values are the transformed, fictitious user responses we use to construct the scenarios. $A_{i}^{\left(k+1\right)}$ is the value of concept $C_{i}$ at timestep k+1 ; $A_{i}^{\left(k\right)}$ is the value of concept $C_{i}$ at timestep *k*; $A_{j}^{\left(k\right)}$ is the value of concept $C_{j}$ at timestep *k*; and $w_{\;ji}$ is the influence of concept $C_{j}$ on concept $C_{i}$ , expressed as a weight. *f* is a threshold function that transforms the content of the function. For all but the three risk assessment parameters, we used one of the most common threshold functions used in FCMs,^41^ the hyperbolic tangent function that transforms the content into the interval [−1,1] :

$$f(x)\;=\;\tan h(x)\;=\;\frac{e^{x}\;-\;e^{-x}}{e^{x}\;+\;e^{-x}}.$$

To prevent the values of the three risk assessment parameters, *Violent loss*, *Recent inpatient treatment*, and *Time since loss*, to change between simulation iterations, we chose the linear activation function for *Time since loss* instead and a bivalent activation function for the two Boolean parameters *Violent loss* and *Recent inpatient treatment*.

$$\begin{array}{rl} \mathrm{Linear}\;f(x) & \;=\;x\\ \mathrm{Bivalent}\;f(x) & =\{\begin{array}{c} -1,\;\;if\;x\leq 0\\ \;\;1,\;\;if\;x>0 \end{array}. \end{array}$$

For the first scenario which represented a user that clearly needs support outside the service, the algorithm's outcome was *Escalate*. The algorithm's outcome for the second scenario which represented a user that could clearly continue using the service by themselves was *Do not Escalate*. For the third scenario, a fictitious user who should seek support, but it shows less obviously than in scenario 1 in their questionnaire responses, the algorithm's decision was *Do not Escalate*. And for the final scenario, a fictitious user who can continue using the service, but it shows less obviously than in scenario 2, the algorithm advised *Escalate*.

### FCM model limitations and suggestions for improvement

Next to configuring the weights of the FCM using the input of clinical experts, the interview data was analyzed with regard to the participants’ appraisal of the FCM as a decision-making algorithm for detecting risk situations in a grief eMental health service. While filling in the FCM weights, the experts participating in this study identified limitations when using the FCM to determine whether or not someone using the online grief self-help service should seek offline support. They also provided suggestions to improve the FCM decision-making algorithm in the future.

Most experts struggled with modeling the relation between *Time since loss* and the two outcomes of the FCM, as well as with modeling its influence on other symptom concepts linearly. They explained that the general expectation regarding *Time since loss* is that “time heals wounds.” The more time has passed, the better one usually copes with a loss. However, if that is not the case, if the loss has occurred long ago and the person still suffers intensely from the loss, then this is an important indicator that someone is stuck in their grief process. In addition, it is not uncommon to see a rise in grief symptoms between the first and the second year after the loss, while, generally, grief symptoms decrease as time passes by.

“Especially if the loss is already quite a long time ago and they still have quite strong grieving symptoms, then I think ‘Yes, now it is time to seek professional help’. To me, this would be one of the most important predictors or indicators for professional help” (Expert 7).

Another limitation of the current FCM is that its decisions are based on one measurement point instead of the history of a user's measurements. The experts in this study explained that to decide whether or not someone should seek offline support, how long symptoms have been elevated is an important decision criterion.

“Regarding the grief symptoms, if it gets worse several times, then I would look at that as an alarm bell. But if, for example, from the first [measurement] to the second they get worse I would perhaps not yet say ‘Please seek professional help’. The self-help modules are very demanding and activate grief. From that point of view, it is also clear to me that they can temporarily deteriorate [the symptoms]” (Expert 1).

A discussion with expert 3 revealed that the influence of the three risk concepts *Violent loss*, *Recent inpatient treatment*, and *Time since loss* is challenging to model using the FCM. In the FCM, relations between symptom concepts can strengthen or weaken the influence of any single concept on the outcome. However, there is no logical influence from the other symptom concepts in the FCM on these three risk concepts. For example, however high someone's grief symptoms are, there is no logical influence on how long ago the loss has occurred. Therefore, the risk concepts do have outgoing influences, but no incoming influences, increasing their overall influence on the model's outcome since the risk concepts cannot be relativized within the FCM. Several experts suggested how the FCM could be extended to better reflect how they make clinical decisions in practice. First, the current selection of FCM concepts overemphasizes a mourner's negative emotions and symptoms, while positive experiences such as reminiscing fond memories are also part of the grieving process.

“You’re actually only checking the negative sides of the person's experience; which is ok, of course, because we are worried and we want to know [about them], but it's not only negative. You remember the positive sides and, you know, there is laughter. Maybe the model indicates that it should be bad all the time, but that is not the case” (Expert 6).

In addition, individual factors such as the mourner's resilience and the significance of the lost relationship for their personal identity play a large role in how well one copes with the loss of a partner. When loss experiences accumulate, which is not uncommon in later life, a person's resilience is compromised. The cumulation of loss experiences is worthwhile to take into account, especially for older mourners:

“There are unprocessed previous losses and that’s what I often encounter with older people. […] They are from a different generation. Regarding loss experiences, things have often remained unmentioned and those things are revived by the most recent loss, in this case, the loss of a partner. This resonates quite a bit” (Expert 5).

Other suggestions for additional symptom concepts include assessing how traumatic the loss experience has been for the mourner, in contrast to focusing on whether or not the loss has been violent and to complement *Hopelessness* with assessing the mourner's feeling of entrapment. A final suggestion was to incorporate different urgency levels when advising someone to seek offline support. Depending on the user's situation, it may be more or less urgent that they seek immediate support and this can be reflected in the recommendation the program gives.

## Discussion

In this article, we present a monitoring module for an online self-help service for older mourners that recommends seeking offline (professional) support when the user's suffering becomes highly disruptive for their everyday life. We present a user profile specific to grief and we configure a FCM decision algorithm. The user profile consists of user parameters relevant for a mental eHealth service targeted at mourning older adults and a measurement protocol to monitor their situation regularly, reflecting Tielman et al.'s^11^ information gathering stage in their two-stage model for detecting risk situations in eMental health. The FCM decision algorithm recommends seeking offline support once a risk has been detected in the user's situation. The FCM represents Tielman et al.'s second phase, the decision-making. The current study demonstrates the configuration of the FCM decision algorithm based on expert knowledge and investigates their performance in four fictitious user scenarios. The developed FCM consists of eight monitoring concepts, including *Grief symptoms*, *Hopelessness*, *Social isolation*, *Therapeutic progress*, *Crisis detection*, *Violent loss*, *Recent inpatient treatment*, and *Time since loss*. The two outcomes of the FCM are either to *Escalate*, meaning that the service recommends the user to seek offline support, or *Do not escalate*, in which case the system encourages the user to continue using the service as is.

### Preliminary FCM algorithm evaluation using scenarios

The four scenarios explored in this paper represent fictitious users and their responses to the monitoring questionnaires, formulated by a clinical expert in grief. The results of the tests show that while the current FCM algorithm distinguishes well between unambiguous cases, it can confuse borderline cases. A notable difference between the unambiguous and the borderline cases in this article is users’ fictitious *Time since loss* responses, as well as whether or not the fictitious user has suffered a violent loss or not, meaning that they lost their spouse due to an accident, suicide, or another form of violent death. The borderline scenarios exemplify the difficulties of modeling the influence of *Time since loss* linearly as the current FCM does. As the experts explained in the interviews, *Time since loss* is an important indicator, but its influence on the model's outcome depends not only on its own value but also on the value of other concepts in the model. According to the experts in this study, “time is generally expected to heal wounds”; however, when this is not the case, it becomes an important decision criterion to decide whether a mourner should consider seeking offline (professional) support. It has a strong positive relation to the outcome *Escalate* and a strong negative relation to the outcome *Do not escalate* if and only if the rest of the symptom concepts indicate that the user is suffering emotionally. Otherwise, the signs of its weights are vice versa and the strength of the relation is lower. Unfortunately, the current FCM does not capture this information because it does not allow for conditional weight setting. It is likely that this led to the incorrect classification of the third scenario as no need to recommend the user to seek support, even though the scenario depicts a user who feels severely impaired in their daily life despite the loss having occurred more than 12 months ago. The second borderline case involves a fictitious user who has suffered a *Violent loss*. In the current weight matrix, *Violent loss* has a very high positive relation, not only to the outcome *Escalate* but also to other parameters (e.g. *Hopelessness* and *Grief symptoms*) that have in turn strong and positive relationships to *Escalate*. In essence, it acts as a magnifier in the model for other symptoms of suffering, such as *Grief symptoms*. This in combination with the difficulties of modeling the conditional influence of *Time since loss* in the current FCM caused the algorithm to be led astray. The FCM decision algorithm is currently further evaluated as part of ongoing evaluation studies^21^ of the online grief service in which it is integrated and it will be interesting to see how it performs in a nonfictitious setting involving older users of the grief service.

### Future FCM algorithm improvements

The above analysis of how the FCM algorithm performed in the scenario experiments exemplifies a strength of using FCMs for detecting risk situations in mental eHealth: its outcomes can be traced back and are thereby explainable. Transparency of decision-making, also referred to as scrutability, is of utmost importance for decision support in healthcare where stakes are potentially high and expert users of DSSs need to accept and rely on the system's advice. Scrutability is a key advantage of fuzzy systems^18,42,43^ and it facilitates identifying approaches to improve the current FCM. For instance, to improve the accuracy of the current FCM in detecting risk situations in borderline cases, the conditional influence of *Time since loss* should be represented in the decision-making. This could be realized by introducing a rule-based extension that adjusts the weight from being positive towards the outcome *Escalate* to negative if the user's suffering, as measured by other symptom concepts in the model such as *Hopelessness* and *Grief symptoms*, stays below a to-be-determined threshold. This approach is similar to the approach taken by Papageorgiou et al.^18^ to account for the differing influences of the concepts psychomotor status and sleep disturbance in detecting depression in an elderly population, depending on the values that these concepts have. For example, in their FCM, psychomotor status has one of two values: psychomotor agitation or psychomotor retardation. Depending on which of the two values is activated, the outgoing weights from the concept psychomotor status to other concepts in the FCM change.

A second approach to improving the current FCM is to scrutinize the weight aggregation process. In the current study, not all relations between symptoms were rated by all experts because their task was to rate the relations that they considered most important. Consequently, some relations between symptoms were rated by the majority of the experts, while others were rated by one or two experts. As a result, individual expert ratings influenced the combined weight matrix to differing degrees. While there is no golden standard for managing expert disagreement during the weight aggregation process, some^34,41^ argue that the effects of unequal numbers of ratings for each relation between symptoms as well as disagreement between experts can be mitigated by any rule-based or mathematical approach that is reasoned properly. For instance, Reckien et al.^44^ suggest a rule-based approach for aggregating highly divergent weights by excluding weights that diverge too much from the arithmetic mean. Another approach could be to eliminate weights from the aggregation that less than a predetermined number of experts have rated. Stylios and Groumpos^41^ apply a credibility factor for regulating how much influence the weights provided by individual experts have in the combined weight matrix. Each expert starts with a credibility factor of 1 with which the suggested weights of the expert are multiplied. The expert is iteratively “penalized” if their suggested weights divert too much from the weights suggested by the rest of the sample.

A final approach to improving the current FCM heeds the recommendation of the experts that participated in this study and involves reconsidering the FCM architecture and introducing more symptom concepts to model the user's resilience to outweigh the current focus on suffering in the FCM. Another way of emphasizing positivity instead of focusing on suffering is to enhance the impact of *Therapeutic progress*, for example, by adding a rule that counteracts an escalation under the condition that the user feels that they are making progress, despite their suffering. In such a situation, the suffering measured in the CRA may be less reason for concern and part of a healthy mourning process. Bourgani et al.^45^ furthermore stress the importance of including temporal information in medical decision-making. The current monitoring module has no means to take the history of the user's monitoring responses into account, while the experts in this study pointed out that this information is important to consider when deciding whether or not a user should seek offline (professional) support. Since including temporal information in FCMs is not straightforward,^34^ information about the duration of the user's suffering could be added via a rule-based extension that takes the user's measurement history into account and formulates rules about when it indicates a risk situation.

The above approaches consider limitations of the current FCM and recommendations for future work to improve its capacity to reliably detect risk situations in an eMental health service to aid older mourners. Tielman et al.^11^ consider another factor that determines the effectiveness of monitoring modules in mental eHealth. Systems that ultimately rely on the user's initiative to seek professional support depend on their motivation to follow-up on the system's recommendation to seek offline support. The authors argue that depending on (a) the user's initial stance on involving professional support (negative, doubtful, and positive) and (b) the severity of the risk situation (severe, negative, and doubtful), the system should adopt different strategies to persuade the user. For example, a user that is doubtful about involving professional support and whose situation is severe needs to be persuaded. On the contrary, a user that is generally positive towards involving a professional and whose situation is severe requires facilitation of the self-referral process. This approach to modeling the user's motivation to accept and act on the system's recommendations is in line with a suggestion from an expert that participated in this study to design different urgency levels for recommendations, depending on the severity of the situation among other factors.

### Limitations

The current study has limitations. First, we gather semiquantitative data via individual interactive sessions with eight clinical experts. Since our approach to aggregating weights is quantitative in nature, the size of the expert sample becomes a point of discussion. There is, however, no standard to determine a sample size for fuzzy cognitive mapping studies. Studies that determine the very concepts of the FCM alongside their weights can use the extent to which the FCM concepts are saturated as indicator that the usefulness of including more experts has been exhausted. We chose the concepts of the FCM based on a previous study, and hence, our concepts were fixed. Olazabal and Reckien^34^ acknowledge that sample sizes differ tremendously between individual studies, ranging from only a few participants (three in Papageorgiou et al.^18^) to studies aggregating up to 376 individual maps.^46^ The authors stress that developing meaningful and usable FCMs is a matter of careful selection of experts rather than sample size. The current study design, individual one-on-one interviews, can be regarded as a second limitation. While fuzzy cognitive mapping studies use a wide variety of individual as well as group elicitation methods,^34^ the experts participating in this study may have profited from a group setting to discuss the weights between FCM concepts and to exchange professional experiences. Experts indicated that determining exact weights for the relations between concepts was challenging. Discussing with peers could have reduced the perceived challenge of the task and limited the extent to which experts disagreed about weights, paving the way to a more coherent distribution of weights. Another argument in favor of a group design is that individual FCMs are inherently subjective with regard to the expert's professional experiences. They are also affected by recency biases, meaning that recent professional experiences may be more salient and, therefore, appear more strongly related to FCM outcomes. Future group discussions could mitigate some of these limitations, while introducing challenges of their own, such as logistical challenges and fostering groupthink.

## Conclusion

The current article presents a monitoring module to detect risk situations in an eMental health self-help service to aid mourning older adults and to encourage users to seek offline support in times of need. The monitoring module uses an automatic FCM decision-making algorithm, configured with the help of eight clinical psychologists. FCMs are a powerful tool for modeling human reasoning due to their capacity to deal with vague definitions of symptoms provided by multiple discipline experts as well as the causal relations between them. Based on four fictitious scenario experiments, the resulting FCM appears to detect clear cases of risk and no-risk, but its accuracy in detecting less clear cases can be increased. To improve the current FCM algorithm, its assumption that symptoms are linearly related to the decision whether or not the user should seek offline support can be improved by introducing rules that regulate the influence of symptoms for which the linearity assumption does not hold, such as *Time since loss*. Another promising approach is to scrutinize the weight aggregation process and to actively deal with experts’ disagreement regarding individual model weights. Due to their scrutability and independence of large amounts of privacy-sensitive patient mental health data for model configuration, FCMs hold much potential for automatic decision-making in eMental health. However, lack of clear guidelines and golden standards for constructing FCMs require future work. The contribution of the current article is showcasing the construction of a FCM decision algorithm for an eMental health service and, in doing so, unraveling challenges when using FCMs for decision-making in eMental health applications.

## Appendix. Monitoring questionnaires

### Initial risk assessment questionnaire (IRA)

Ratings in brackets

Time passed since spousal loss

1. How much time has passed since you lost your partner?

□ Less than a month (0)
□ Between 1 to 6 months (1)
□ Between 7 to 12 months (2)
□ More than 12 months ago (3)

Characteristics of the loss

2. Did the circumstances of your loss involve any of the following

□ Accident
□ Suicide
□ Violence
□ Yes (1)
□ No (0)

Inpatient treatment

3. Have you received treatment for a psychological condition in an inpatient clinic in the past?

□ Yes, in the past year (1)
□ Yes, more than a year ago (0)
□ No (0)

Technical skills

4. How would you describe your skills with a computer and the internet? (*Ratings in parentheses*)

□ Very poorly, I avoid using the computer as much as possible. (0)
□ Poor, I often need assistance to find my way around the computer and the internet. (1)
□ Fair, I can perform standard actions on the computer *without* assistance, for example sending and reading e-mails, printing, or managing my bank account online. (2)
□ Good, I perform standard actions regularly on the computer and occasionally use it for other purposes (e.g., playing games, booking a vacation, online shopping. (3)
□ Very good, I find my way around the computer and the internet very easily and use both regularly for standard business and other purposes. (4)

Suicidality

1. In the past 2 weeks, I have contemplated to commit suicide.

□ Yes (1)
□ No (0)

**If yes**, adapted Scale for Suicide Ideation (SSI) items 12 through 16 assess plans, preparations, and resolve to make a suicide attempt:

2. In the past 2 weeks, I have considered a plan for a suicide attempt.

□ No, I have not worked out a plan. (0)
□ Yes, but the details of the plan are not worked out. (1)
□ Yes, and the details of the plan are well worked out. (2)

3. In the past 2 weeks, I felt that there was an opening for a suicide attempt or a suicide plan that I thought about was available.

□ The contemplated plan is not available; there is no opportunity. (0)
□ The contemplated plan would take time and/or effort and it is not readily available. I don’t see an opportunity. (1)
□ The contemplated plan is available or there is another opportunity to commit suicide. (2)
□ I anticipate that in the future, the contemplated plan will be available or there will be an opportunity. (2)

4. In the past 2 weeks, I have considered how capable I am to commit suicide.

□ I am certain that I have **no** courage and/or **no** competence to commit suicide. (0)
□ I am uncertain of my courage and/or competence to commit suicide. (1)
□ I am certain that I have courage and/or competence to commit sucide. (2)

5. In the past 2 weeks, I have engaged in actual preparation for the contemplated suicide plan.

□ No, I did no preparation at all. (0)
□ I started to prepare the contemplated suicide plan. (2)
□ I have completed preparing the contemplated suicide plan. (2)

Continuous risk assessment questionnaire (CRA)

Crisis detection

1. In the past 2 weeks, how often have you felt impaired in your daily life or experienced severe emotional distress which made it difficult to think of other things?

□ Not at all
□ A few days
□ More than half the days
□ (Nearly) every day

2. In the past 2 weeks, how often have you felt that you need extra support to cope with everyday life or getting through the day?

□ Not at all
□ A few days
□ More than half the days
□ (Nearly) every day

Depressive symptom: Hopelessness

1. In the past 2 weeks, how often have you given up because your future feels dark, and seems to only get darker?

□ Not at all
□ A few days
□ More than half the days
□ (Nearly) every day

2. In the past 2 weeks, how often have you felt that all that awaits you in your future is emptiness, loneliness or suffering and that there is nothing you can do about it?

□ Not at all
□ A few days
□ More than half the days
□ (Nearly) every day

Grief symptoms

1. In the past 2 weeks, how often have you felt impaired in your daily activities by intense feelings of emotional pain or suffering related to your grief?

□ Not at all
□ A few days
□ More than half the days
□ (Nearly) every day
□ Not at all
□ A few days
□ More than half the days
□ (Nearly) every day

2. In the past 2 weeks, how often have you felt impaired in your daily activities by feeling stunned or dazed by your loss?

Suicidality

1. In the past 2 weeks, I have contemplated to commit suicide.

□ Yes
□ No

**If yes**, adapted Scale for Suicide Ideation (SSI) items 12 through 16 assess plans, preparations, and resolve to make a suicide attempt:

2. In the past 2 weeks, I have considered a plan for a suicide attempt.

□ No, I have not worked out a plan.
□ Yes, but the details of the plan are not worked out.
□ Yes, and the details of the plan are well worked out.

3. In the past 2 weeks, I felt that there was an opening for a suicide attempt or a suicide plan that I thought about was available.

□ The contemplated plan is not available; there is no opportunity.
□ The contemplated plan would take time and/or effort and it is not readily available. I don’t see an opportunity.
□ The contemplated plan is available or there is another opportunity to commit suicide.
□ I anticipate that in the future, the contemplated plan will be available or there will be an opportunity.

4. In the past 2 weeks, I have considered how capable I am to commit suicide.

□ I am certain that I have **no** courage and/or **no** competence to commit suicide.
□ I am uncertain of my courage and/or competence to commit suicide.
□ I am certain that I have courage and/or competence to commit sucide.

5. In the past 2 weeks, I have engaged in actual preparation for the contemplated suicide plan.

□ No, I did no preparation at all.
□ I started to prepare the contemplated suicide plan.
□ I have completed preparing the contemplated suicide plan.

Social isolation

1. In the past 2 weeks, how often have you been thinking that the people in your life would be better off if you were gone?

□ Not at all
□ A few days
□ More than half the days
□ (Nearly) every day

2. In the past 2 weeks, I have avoided getting in touch with friends and family.

□ Not at all
□ A few days
□ More than half the days
□ (Nearly) every day

Therapeutic progress

1. In the past 2 weeks, I have had positive experiences in addressing my problems and/or I have gained important insights.

□ Very strongly disagree
□ Disagree
□ Agree
□ Very strongly agree

2. In the past 2 weeks, I generally have been feeling better.

□ Very strongly disagree
□ Disagree
□ Agree
□ Very strongly agree
